# Phytochemical, in-vitro biological and chemo-preventive profiling of *Arisaema jacquemontii* Blume tuber extracts

**DOI:** 10.1186/s12906-019-2668-4

**Published:** 2019-09-14

**Authors:** Saira Tabassum, Muhammad Zia, Esperanza J. Carcahe de Blanco, Riffat Batool, Roohi Aslam, Sajid Hussain, Qamar Wali, Muhammad Mudassar Gulzar

**Affiliations:** 1 NUTECH School of Applied Sciences and Humanities, National University of Technology (NUTECH), Islamabad, Pakistan; 2 Department of Biotechnology, Quaid-I-Azam University, Islamabad, 45320 Pakistan; 3 Division of Pharmacy Practice Science and Division of Medicinal Chemistry and Pharmacognosy, College of Pharmacy, The Ohio State University, Columbus, OH USA

**Keywords:** Phenolic and flavonoid content, Antioxidant activities, Antimicrobial, SRB cytotoxic assay, NF-kB pathway

## Abstract

**Background:**

*Arisaema jacquemontii* is traditionally used in treatment of different diseases. In this study, phytochemical, in vitro biological and chemo-preventive screening of *A. jacquemontii* was carried out to explore its pharmacological potential.

**Methods:**

The dried tuber of *A. jacquemontii* was extracted in 11 organic solvent mixture of different polarity. The extracts were screened for phytochemical assays (phenolics and flavonoids), antioxidants potential (free radical scavenging activity, total antioxidant activity, reducing power), biological activities (antibacterial, antifungal, cytotoxic, antileishmanial, protein kinase inhibition), and chemopreventive activities using different cell lines through standard protocols.

**Results:**

Significant amount phenolic contents were determined in EtOH and MeOH extracts (210.3 ± 3.05 and 193.2 ± 3.15 μg GAE/mg, respectively). Maximum flavonoid content was determined in MeOH extract (22.4 ± 4.04 μg QE/mg). Noteworthy, DPPH scavenging activity was also recorded for MeOH extract (87.66%) followed by MeOH+EtOAc extract (85.11%). Considerable antioxidant capacity (7.8 ± 0.12 μg AAE/mg) and reducing power (3.1 ± 0.15 μg AAE/mg) was observed in extract of MeOH. The LC_50_ against brine shrimp and leishmanial parasite was found 9.01 and 12.87 μg/mL for n-Hex and CHCl_3_ extracts, respectively. The highest zone of inhibition against *Streptomyces* hyphae formation (12.5 ± 1.77 mm) by n-Hex extract. Growth zone of inhibition 13.8 ± 1.08 mm was recorded for EtOAc and MeOH extracts, respectively against *Micrococcus luteus* while 10.0 ± 0.11 mm for MeOH extract against *Aspergillus flavus*. In-vitro cytotoxic assay showed that n-Hex extract had higher cytotoxicity against DU-145 prostate cancer and HL-60 cancer cell lines. NF-kB and MTP potential showed 34.01 and 44.87 μg/mL for n-Hex and CHCl_3_ extracts, respectively in chemo-preventive potential.

**Conclusion:**

The study concludes that *Arisaema jacquemontii* bears significant phytochemical activity and pharmacological activities, this plant can be further explored for isolation of active component against a number of aliments.

## Background

The significance of traditional health systems and medicinal plants cannot be denied because nature has placed cure against many diseases in natural materials. Therefore interest towards the herbal medicines and isolation of active compounds is growing incredibly. Medicinal plants are being practiced for different ailments in most of the developing countries. Thousands of plant species have been used for health care purposes in various cultures and derivations of medicinal preparations [1]. A variety of drugs have been isolated from plants, i.e. aspirin from *Filipendula ulmavia*, benzoin from *Slyrax tonkinensis,* morphine from *Papaver somniferum*, quinine from *Cinchona pubescens,* vincristine and vinblastine from *Catharanthus roseus* [2]*.* Despite of the rapid progression toward the field of medicinal chemistry, resistance of microbes against therapeutic agents and enhanced exposure to free radicals necessitate the search of novel therapeutic agents from medicinal plants [3–5].

*Arisaema jacquemontii* (Araceae) herb (local name “Sap-ki-booti”) is freely available in northern region of Pakistan. *A. jacquemontii* belongs to genus *Arisaema* which constitutes more than 250 species. It is a deciduous perennial herb that grows to a height of about 1–2 m [6] and is further distributed in many parts of East Asia, Afghanistan to South East Tibet, upper and lower alpine zones in the dried areas of Himalayas. Various constituents of this plant, especially tuber lectins are being used for medicinal purposes [7]. A tuber lectin purified from this plant has been reported to exhibit potent anti-insect and anti-proliferative activities [8]. A large number of *Arisaema* species have been reported as anticonvulsants [9]. The rhizomes or tubers of *A. calcareum*, *A. serratum*, *A. asperatum, A. heterophyllum*, and *A. amurense* are documented as analgesic, antitumor and pesticidal potential [10]. In addition, secondary metabolites (phenolic acids, flavonoids, anthocyanidins and tannins) isolated from these plants have shown remarkable antioxidant and immunomodulatory activities [11, 12]. Synergistic effect of drugs and/or herbal extracts can be beneficial or harmful. In case of herbal extracts it depends upon the cumulative effect of active ingredients resulting in similar or related outcomes. For example, vitamin E is an antioxidant and vitamin C might help to recycle oxidized vitamin E into active vitamin E, thus, a synergistic [13]. The different petrochemicals were isolated from the medicinal plants e.g. β-sitosterol was isolated from medicinal plant having significant potentials against the *L. tropica* KWH23 by in-vitro anti-promastigotes. β-sitosterol mediated leishmanicidal potential via apoptosis confirmed by DNA interaction and molecular docking studies [14].

In continuation of efforts to verify the efficiency of medicinal plants with traditional and local knowledge, *Arisaema jaquamontii* tuber was extracted using polar to non-polar solvents. The extracts were pharmacologically evaluated by using cost effective and highly efficient assays involving antileishmanial, brine shrimp cytotoxicity, SRB cytotoxicity and chemo preventive potential of NF-kB pathway and MTP cycle, protein kinase inhibition, antibacterial and antifungal assays. This study identifies the medicinal potential of A. *jaquamontii* that will lead towards isolation of active constituents for evaluation in drug usage.

## Methods

### Plant material

*A. jacquemontii* was collected in the month of July from Lakary mountains, Shamshaki, District Karak, Khyber Pakhtunkhwa, Pakistan. The plant material was authenticated by Prof Dr. Mir Ajab Khan, Department of Plant Sciences, Quaid-i-Azam University Islamabad, Pakistan. A voucher specimen (PHM: 491) was deposited in the Herbarium of Medicinal Plants, Quaid-i-Azam University Islamabad, Pakistan.

### Chemical and reagents

The study involved the use of analytical grade solvents which were purchased from Sigma (Sigma-Aldrich USA) i.e. n-hexane (n-Hex); Chloroform (CHCl_3_); Acetone (Ace); Ethyl acetate (EtOAc); Ethanol ((EtOH); Methanol (MeOH) and Dimethyl sulfoxide (DMSO). The reagents; Gallic acid (G7385); Quercetin (Q4951); 2,2-diphenyl-1-picryl-hydrazyl (Cat#757621; DPPH); Potassium acetate; Aluminum Chloride; Folin-ciocaltau (F-C) reagent -Sodium carbonate; Ascorbic acid (A5960); Ammonium molybdate; Sodium phosphate; Sulfuric acid; Ferric cyanide; Trichloroacetic acid and Potassium ferricyanide were procured from Merck (Merck KGaA, Germany). Roxithromycin (R4393); Cefixime (1097658); Terbinafine (T8826), Doxorubicin (D1515), Taxol (T1912) and Amphotericin-B (A2942) was obtained from Sigma (Sigma-Aldrich USA) were used Reference standards in the current study.

### Preparation of extracts

Tubers of *A. jacquemontii* were washed with tap water then rinsed with distilled water. The material was air dried under shade at room temperature. After comminuting the plant material by using the commercial miller to coarse powder, 10 g of dried powder material was soaked separately in beakers, each containing 40 mL of extraction solvents of ranging polarity from highly non-polar to highly polar solvents including n-hexane (n-Hex), Chloroform (CHCl_3_), Acetone (Ace), Ethyl acetate (EtOAc), Ethanol (EtOH), Methanol (MeOH). Along with individual solvents, combinations of solvents at 1:1 were also used as follow: EtOH+CHCl_3_, MeOH+CHCl_3_, Ace+EtOAc, EtOH+EtOAc, and MeOH+EtOAc. The plant material was soaked for 48 h, thereafter filtered through Whatman No.1 filter paper. The residue was again dipped in respective solvent/s and this process was repeated thrice. The respective extracts were combined and concentrated by evaporating the solvent under reduced pressure in a rotary evaporator (Buchi, Switzerland) at 45 °C.

### Phytochemical screening

#### Determination of total phenolic contents

The total phenolic contents of the extracts were determined by Folin–Ciocalteu method [15]. Briefly, stock solutions of extracts (4 mg/mL) were prepared in DMSO and 20 μL of each extract was transferred to each well of 96 well plate. The solutions were then mixed with 90 μL of F-C (Folin–Ciocalteu) reagent. After 5 min, the reaction mixture was mixed with 90 μL of Na_2_CO_3_ solution (7.5%). The reaction mixture was incubated for 1 h and absorbance was measured at 650 nm by using microplate reader (Bioteck). Blank (DMSO) and standard (gallic acid in DMSO) were run simultaneously. The resultant TPC is calculated as μg gallic acid equivalents per mg extract (μg GAE/mg extract).

#### Determination total flavonoid content (TFC)

The TFC of the extracts was determined by aluminum chloride method as reported previously [15]. Briefly, extract solution (20 μL, 4 mg/mL DMSO) was mixed with 10 μL of aluminum chloride (10%) and 10 μL of potassium acetate (1 M). Subsequently, distilled water was added to get a final volume of 200 μL. After 30 min of incubation (Incubator IC83 Yomato, Japan), absorbance was measured by using microplate reader (Bioteck) at 415 nm at 37 °C. Quercetin was used as standard flavonoid and results are expressed as μg quercetin equivalent per mg extract (μg QE/mg extract).

### Biological evaluation

#### DPPH free radical scavenging assay

2, 2-diphenyl-1-picrylhydrazyl reagent was employed for the determination of free radical scavenging activity of the extracts [15]. Briefly, stock solutions of extracts (4 mg/mL) were prepared in DMSO. Aliquot of 10 μL of each extract was mixed with 190 μL of DPPH (0.004%). The reaction mixture was incubated in the dark for 1 h. The optical density was measured at 515 nm using microplate reader (Bioteck). Ascorbic acid was employed as positive standard while DMSO as negative control. The extracts were first screened at final concentration of 200 μg/mL and those exhibiting good quenching activity (≥ 50%) were tested at lower concentration to find IC_50_ values. Percent inhibition was calculated by the following formula:

$$ \mathrm{Percent}\ \mathrm{inhibition}\ \mathrm{of}\ \mathrm{the}\ \mathrm{test}\ \mathrm{sample}=\left[\%\mathbf{scavenging}\ \mathbf{activity}=\right(\mathbf{1}-{\mathbf{Ab}}_{\mathbf{s}}/{\mathbf{Ab}}_{\mathbf{c}\Big)}\ast \mathbf{100}\Big] $$

Where Ab_s_ is the absorbance of DPPH solution with sample, whereas Ab_c_ indicates the absorbance of negative control (containing the reagent except the sample). The IC_50_ was calculated by using Table curve software 2D version 4.

#### Determination of total antioxidant capacity (TAC)

Total antioxidant activity of extracts was evaluated following the methodology previously reported [15]^.^ Mixing 100 μL of stock solution of each extract (4 mg/mL in DMSO) with 900 μL reagent solutions comprising of 0.6 M sulfuric acid, 4 mM ammonium molybdate and 28 mM sodium phosphate was done. The reaction mixtures were incubated at 95 °C for 90 min. After incubation the reaction mixtures were cooled down at room temperature and absorbance of each extract was measured at 695 nm by using micro plate reader. A typical blank containing DMSO (100 μL) was used as control. For the calibration curve, ascorbic acid was used at different concentrations with DMSO. The resultant TAC is expressed as μg ascorbic acid equivalent per mg extract (μg AAE/mg extract).

#### Reducing power assay

The reduction potential of the test samples were investigated according to the procedure described previously [15]. Briefly, 100 μL of each sample (4 mg/mL extract in DMSO) was mixed with 200 μL of phosphate buffer (0.2 M, pH 6.6) and 250 μL of 1% potassium ferricyanide solution. The resulting mixtures were incubated for 20 min at 50 °C. After incubation, the reaction mixtures were acidified with 200 μL of 10% trichloroacetic acid. The resultant mixtures were centrifuged at 3000 rpm for 10 min. Supernatant layer (150 μL) was mixed with 50 μL of 0.1% ferric chloride solution in a separate tube and optical density was measured at 630 nm using microplate reader. Ascorbic acid was maintained as positive control and results are expressed as μg ascorbic acid equivalent per mg extract (μg AAE/mg extract).

#### Brine shrimp lethality assay

The degree of lethality to brine shrimps was determined by following previously described protocol [16]. Stock solutions (100 mg/mL) of each sample were prepared in DMSO. *Artemia salina* (brine shrimp) eggs (Ocean Star, USA) were hatched in a bi-partitioned tank filled with artificial sea water (3.8% Sea salt supplemented with 6 mg/mL dried yeast, pH 7). The larger compartment was covered with aluminum foil while the smaller compartment was illuminated with a light source. After 24–48 h incubation period the phototropic nauplii were harvested by using micro pipette and transferred to 96 well plate. Various sub dilutions of 100 mg/mL DMSO stock solution of each extract were tested for lethality determination at final concentrations of 250, 125, and 62.5 μg/mL. The corresponding micro liter of each dilution was transferred to each well containing 10 nauplii and 300 μL sea water supplemented with dried yeast (6 mg/L). Negative control vial included DMSO, nauplii and sea water, but no sample, whereas positive control included 4 mg/ml standard drug doxorubicin, nauplii and sea water. After 24 h incubation period, dead nauplii were counted in each vial and LC_50_ was measured accordingly by comparing percentage mortality with standard drug using Table curve software 2D version 4.

#### Protein kinase inhibition assay

*Streptomyces* 85E strain was used for protein kinase inhibition assays following the published protocol [17]. The microorganism was refreshed in sterile Trypton Soy broth (Merck, Germany) for 24–48 h. The cultured was inoculated ISP4 mineral medium in petri plates. 5 mm Whattman filter paper discs impregnated with 5 μL of 20 mg/mL extracts were placed on seeded plates. The plates were incubated at 28 °C for 72 h. The bald zones of hyphae formation inhibition were measured. Surfactin was used as positive control while DMSO impregnated discs were included as negative control in order to confirm the non toxic effect of DMSO.

#### In vitro antileishmanial activity

In vitro antileishmanial activity was performed with *Leishmania tropica* khw23 strain [18]. The Leishmanial parasite was kindly provided by Dr. Gul Shahnaz and protocol was followed as described [18]. The parasite was cultured in M199 media supplemented with 10% Fetal Bovine Serum at 24 °C.The culture (promastigotes) was harvested at concentration of 1 × 10^6^ cells/mL. Stock solution of each test samples was prepared in DMSO (20 mg/mL) and serially diluted in 96 well plate. The anti leishmanial activity of each sample was determined at concentrations ranging from 62.5–250 μg/mL. The cultured plates, inoculated with parasite and test samples, were incubated at 24 °C for 72 h. Amphotericin-B was employed as positive control while distilled water as a negative control. The live promastigotes were counted under light microscope using improved Neubauer chamber. The data obtained was then statistically analyzed and LC_50_ was estimated using table curve 2D Ver.4 software.

#### Antibacterial activity

Antibacterial assay was performed against five bacterial strains (two Gram positive (*Micrococcus luteus* ATCC# 10240 and *Staphylococcus aureus* ATCC# 6538) and three Gram negative (*Bordetella bronchiseptica* ATCC# 4617, *Salmonella typhimurium* ATCC# 14028 and *Enterobacter aerogenes* ATCC# 13048) following the standard protocol [15]. The strains were purchased from ATCC culture bank and maintained in the lab. The strains were recultured by inoculating them from stored slants onto nutrient broth media and incubated for 18–24 h. The refreshed inoculum was then swabbed onto petri plates filled with nutrient agar. Sterilized deionized water was used to adjust the turbidity to 10^4^ CFU/mL by comparing with McFarland 0.5 BaSO_4_ turbidity standard. The extract (5 μL of 20 mg/ml DMSO) infused filter paper discs (6 mm) were placed on swabbed nutrient agar plate. Roxithromycin (4 mg/ml), Cefixime-USP (4 mg/ml) and DMSO impregnated discs were included as positive and negative control, respectively. After 24 h incubation period, clear zone of growth inhibition around sample and standard treated discs was measured by using vernier caliper. The samples having ≥11.0 mm zone of inhibition were tested at lower concentrations (100–50 and 25 μg/ml) by using microtiter plate broth dilution method to find minimum inhibitory concentration (MIC).

#### Antifungal activity

Antifungal activity of each sample was evaluated following the method described previously [17]. The fungal strains were kindly provided by First Culture Bank of Pakistan Punjab University Lahore Pakistan. Fungal cultures (*Aspergillus fumigatus* FCBP# 66, *Fusarium solani* FCBP# 0291, *Mucor* specie FCBP# 0300, *Aspergillus flavus* FCBP# 0064 and *Aspergillus niger* FCBP# 0198) were cultured on Sabouraud Dextrose Agar medium (SDA; Merck Germany). Prior to the sensitivity determination, the spores of fungal strains were harvested in 0.02% between 20 solution and their turbidity was adjusted according to McFarland 0.5 turbidity standard. Then 100 μL of each harvested spores was swabbed on plates containing SDA. Filter paper discs loaded with 5 μL of extract (20 mg/ml in DMSO) as well as 2.5 μL of standard antifungal terbinafine (4 mg/ml) were placed on swabbed SDA plates. The plates were incubated at 28 °C for 24 h. After that the clear zones of growth inhibition around sample infused discs were measured using vernier caliper.

#### SRB cytotoxic assay against DU-145, HL-60 cancer cell lines

The cytotoxic potential of test samples to adherent cancer cell lines was determined by using SRB colorimetric assay [17]. All the cell lines were cultured in DMEM medium except for HL-60 that was cultured in RPMI-1640. Basic media were supplemented with 5% FBS and 1% antibody (AB) solution and maintained at 37 °C in 5% CO_2_ incubator to achieve the desired level of confluence. For adherent cells, after removing the old medium, cells were washed thrice with PBS and 1 mL of trypsin was added and put in the incubator for 5 min. Cells with trypsin were shaken along with 5 mL of fresh medium and transferred a falcon tube. For suspended cultures, cells were transferred to falcon tube and centrifuged for 5 min at 1500 rpm. Old medium was then replaced with 5 mL fresh medium. Cells were counted using Hemocytometer and seeding density was adjusted to 5 × 10^4^ cells/mL.

During assay, 10 μL of extract was thoroughly mixed with 190 μL of culture in 96-well plate and incubated for 72 h at 33 °C. Then, 100 μL of 20% w/v TCA was added and incubated for 30 min at 4 °C to fix the cells. It was then washed three times with water, air dried and incubated with 100 μL of 0.4% w/v SRB in 1% acetic acid for 30 min at room temperature. Afterwards, wells were washed with 1% acetic acid, air dried and exposed to 200 μL of 10 mM Tris base for 5 min in the plate shaker. Absorbance of the microplate was measured at 492 nm using the plate reader. Taxol (400 μg/mL) was used as positive control while culture media having no visible growth was used as blank. 10% DMSO PBS used was negative control.

### Cancer chemo preventive bioassays

#### Inhibition of TNF-α activated nuclear factor-kappa B (NF-ĸB) assay

The NF-κB assay was carried out in accordance to a previously published protocol [17]. In brief, the Hela cells nuclear extract was treated with each extract and TNF-α was used for evaluation for specific binding. After treatment with test samples, cells were treated with TNF-α (10 ng/mL) and the nuclear extract from the cells extracted using NE-PER™ Nuclear and Cytoplasmic Extraction Reagents kit from Thermo Scientific (Pierce Biotechnology, Rockford, IL, USA). The specific binding ability of activated NF-κB p65 to the biotinylated-consensus sequence under the presence of test samples was assessed using the Pierce™ NF-κB p65 Transcription Factor Assay kit from Thermo Scientific (Pierce Biotechnology, Rockford, IL, USA). The binding activity was measured by detecting the chemiluminescent signal in a Fluostar Optima plate reader. The test samples exhibiting over 50% inhibition at 50 μg/mL were also tested at four concentration levels (0.05 μg/mL – 50 μg/mL), to obtain inhibitory concentration (IC_50_). Rocaglamide was used as a positive control and untreated cells as negative control.

#### Mitochondria transmembrane potential (MTP) assay

Variations on the mitochondria trans-membrane potential were noticed and measured by a fluorescence cell based assay as described previously [17]. In short, cells were cultured in black and clear bottom plates of 96-well plates at a density of 6 × 10^4^ cells/mL and incubated for 24 h at 37 °C in a humified 5% CO_2_ incubator. Cells were then treated with the test samples and staurosporine (positive control) for 2 h. The cells were incubated with the lipophilic cationic dye (5, 5′, 6, 6′-tetrachloro-1, 1′, 3, 3′-tetraethyl benzymidazolyl carbocyanide) JC-1 for 30 min. After incubation, cells were washed with a buffer for removing unbound staining reagent. The clear bottom plates were then scanned with fluorescence imaging microscope. Black 96-well plates were analyzed by a plate reader (FLUO-star-Optima-fluorescence) with excitation and emission wavelengths of 485 nm and 530 nm respectively for JC-1 monomers while excitation and emission wavelengths of 560 nm and 595 nm respectively for J-aggregates. Measurements were performed in triplicate and are representative of at least two independent experiments.

### Statistical analysis

The data is presented as mean ± standard deviation (SD). All the experiments were carried out in triplicate. SPSS Ver. 21 software was used for Post Hoc Multiple Comparison test in One Way ANOVA and IC_50_ was determined by using Table curve software 2D version 4. Origin 8.5 software was used for graphical presentation.

## Results

Total 11 extracts of *A. jaquamontii* tubers were prepared in different solvents and used for determination of pharmacological potential. The percentage yield of extracts along with sample codes is described in Table 1. Maximum extraction yield was obtained with the MeOH followed by MeOH+CHCl_3_ and EtOH. Extract of n-Hex was obtained in minimum quantity.

**Table 1:** *A. jacquemontii* extraction in different organic non polar to polar solvent with percent yield

S/ No.	Sample code	Solvent	% Yield (g)
1	n-Hex	n-hexane	0.289
2	CHCl_3_	Chloroform	0.55
3	Ace	Acetone	0.321
4	EtOAc	Ethyle acetate	0.765
5	EtOH	Ethanol	6.68
6	MeOH	Methanol	8.753
7	EtOH+ CHCl_3_	Ethanol+Chloroform	2.75
8	MeOH+ CHCl_3_	Methanol+Chloroform	7.159
9	Ace+EtOAc	Acetone+Ethyl acetate	0.589
10	EtOH+EtOAc	Ethanol+Ethyl acetate	1.016
11	MeOH+EtOAc	Methanol+Ethyl acetate	0.975

### Phytochemical evaluation

**Fig. 1:**
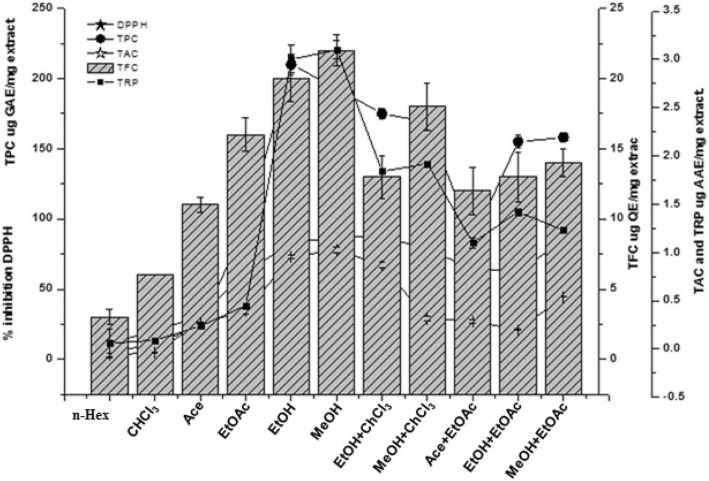
Total phenolic, flavonoid contents and antioxidant activity of different non polar to polar solvent extracts of *A. jacquemontii*

Among all extracts, the highest amount of gallic acid equivalent TPC was determined in EtOH (210.66 ± 4.04 μg/mg) and MeOH extract (193.33 ± 5.40 μg/mg) followed by MeOH+CHCl_3_ (169.66 ± 4.50 μg/mg). The lowest value of TPC was determined in n-Hex extract (6.23 ± 1.40 μg GAE/mg) as shown in Fig. 1. The quercetin equivalent total flavonoid content was found varying between 11.29–22.33 μg/mg. The highest content was observed in MeOH (22.33 ± 3.05 μg/mg), EtOH (20.62 ± 3.15 μg/mg) and MeOH+CHCl_3_ extract (18.30 ± 2.51 μg/mg) (Fig. 1).

### Biological evaluation

The free radical quenching results are summarized in Fig. 1. The DPPH scavenging potential of extracts ranged from 21.61 to 87.66%. Significant activity (*p* < 0.05) was observed for MeOH (87.66%: IC_50_ = 45 μg/ml), MeOH+EtOAc (85.03%: IC_50_ = 76 μg/ml) and EtOH extract (83.11%: IC_50_ = 32 μg/ml). The lowest scavenging activity was found in n-Hex extract with only 12% free radical scavenging potential. The highest antioxidant capacity, expressed as equivalent of ascorbic acid, was observed for MeOH (7.86 ± 0.12 μg AAE/mg) followed by EtOH (7.3 ± 0.15 μg AAE/mg), EtOH+CHCl_3_ (6.7 ± 0.22 μg AAE/mg) and MeOH+EtOAc (4.45 ± 0.20 μg AAE/mg). The lowest TAC was manifested by n-Hex extract (0.36 ± 0.20 μg AAE/mg). The color of the reaction mixture changes either to green or blue depending on the reducing power of the extracts. A high absorbance confirmed higher reducing power. Among all samples, MeOH and EtOH extracts exhibited highest reduction potential i.e. 3.1 ± 0.14 and 3 ± 0.15 μg AAE/mg, respectively (Fig. 1). The least possible reducing power was exhibited by n-Hex extract (0.06 ± 0.057 μg AAE/mg).

### Cytotoxicity assay

**Fig. 2:**
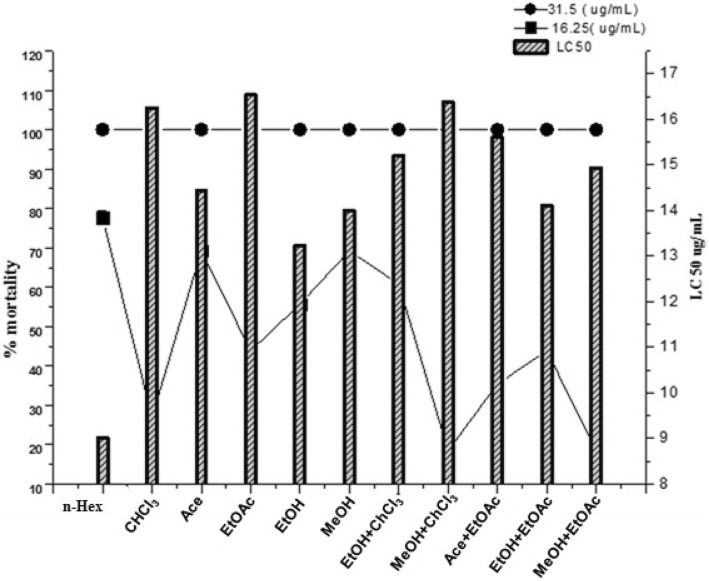
Percent mortality with corresponding LC_50_ (μg/ml) of different non polar to polar solvent extracts of *A. jacquemontii* against brine shrimps larvae

**Fig. 3:**
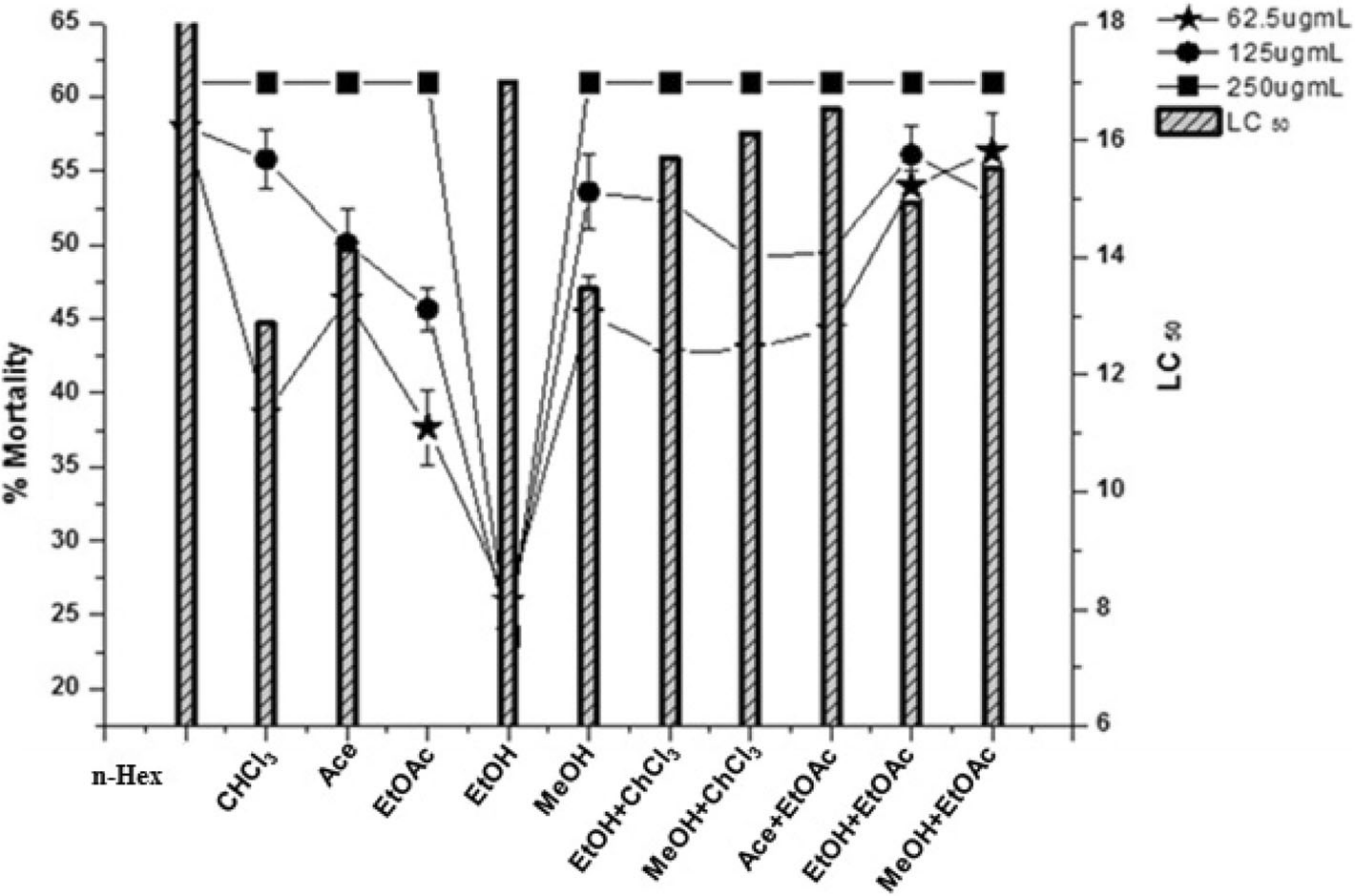
Protein kinase inhibition potential of *A. jacquemontii* extracts. Values are presented as Mean + SD, LSD test for pair wise comparison, not significantly different (*P* > 0.05). GOI of standard Surfactin comes out to be 17 ± 1.2 mm

The percentage mortality/cytotoxicity of the extracts is shown in Fig. 2. LC_50_ values calculated for each extract against brine shrimps ranged from 9 to 18 μg/mL with highly potent cytotoxicity exhibited by n-Hex (80%; LC_50_ = 9.01 μg/mL) and EtOH (67%; LC_50_ = 13.24 μg/mL), MeOH extracts (75%; LC_50_ = 14 μg/mL) and EtOH+EtOAc (60%; LC_50_ = 14.11 μg/mL). Protein kinase inhibitory activity of different solvent extracts was evaluated against *Streptomyces* strain. All samples exhibited significant kinase inhibition potential. Highly significant activity (*p* < 0.05) was recorded for n-Hex extract with 12.5 ± 1.77 mm bald zone of hyphae formation inhibition (Fig. 3).

**Fig. 4:**
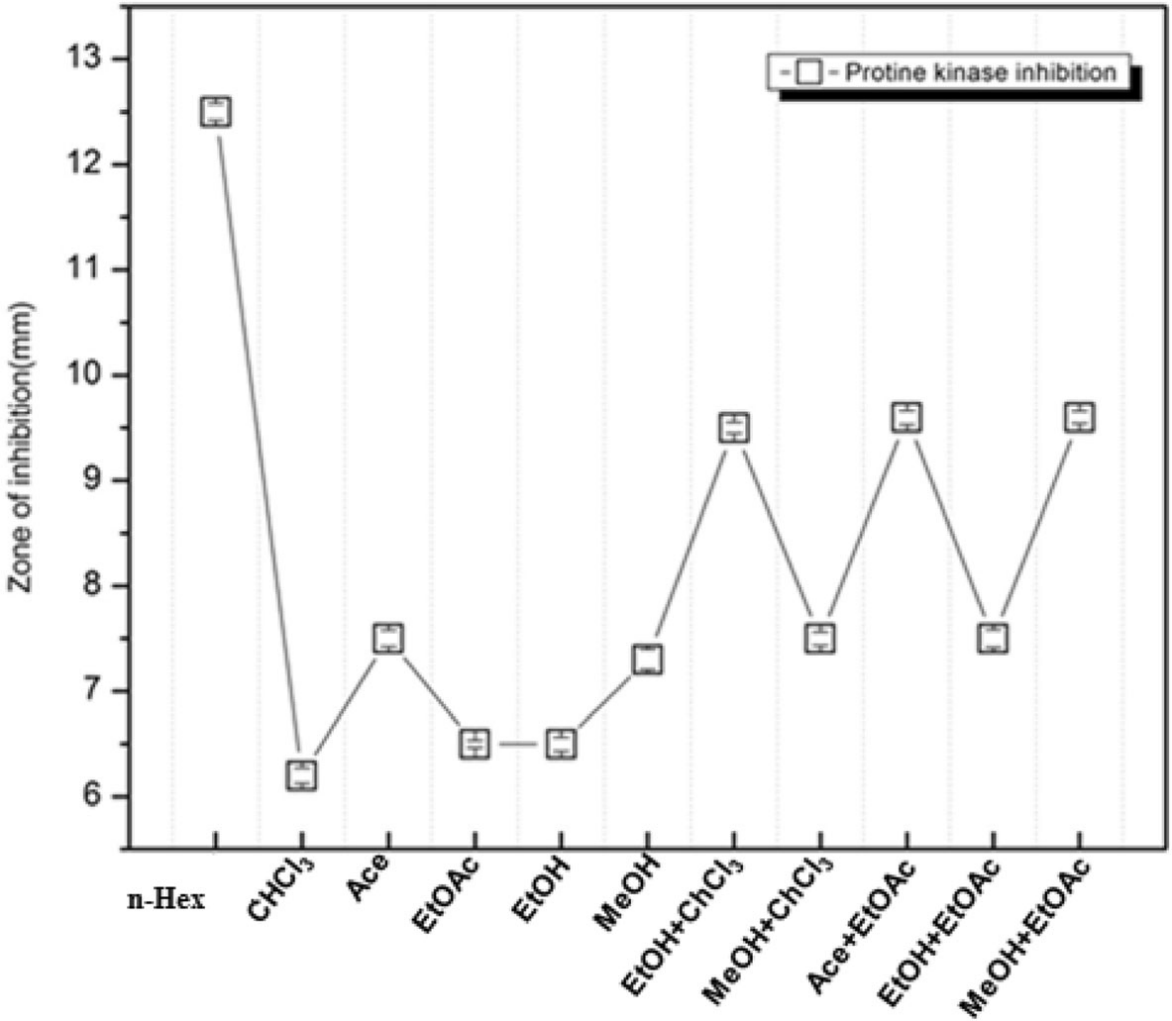
Percent mortality (LC_50_μg/ml) exhibited by different solvent extracts against *L. tropica*

In vitro anti-leishmanial activity of different solvent extracts was studied against *L. tropica* strain (kwh23). The percent mortality of the *L. tropica* is depicted in Fig. 4. The results reveal that all the plant extracts exhibited significant anti-leishmanial activity with 100% mortality at highest concentration (250 μg/mL), except for EtOH extract (76.33%). As the concentration decreased, the percent mortality reduced. At concentration 125 μg/mL, the highest activity was observed for CHCl_3_ extract (82.33%; LC_50_ = 12.87 μg/mL), MeOH extract (78%; LC_50_ = 13.47 μg/mL) and Ace extract (74%; LC_50_ = 14.13 μg/mL).

### Antimicrobial activity

**Fig. 5:**
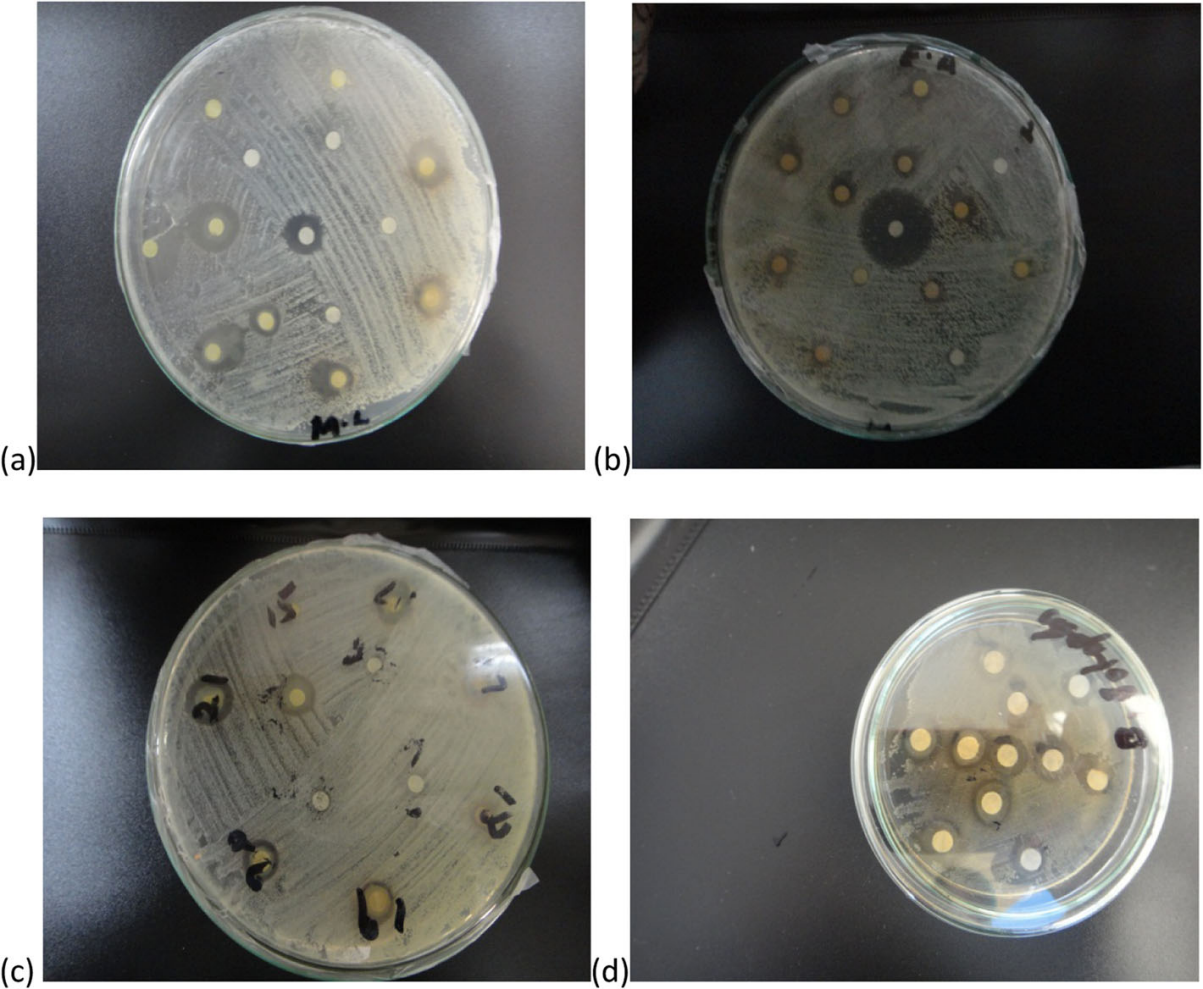
Antibacterial activity images of *A. jacquemontii* extracts against **a** *M. lutus* **b** *E. aerogens* **c** *S. aureus* **d** *B. bronchiseptica*

**Fig. 6:**
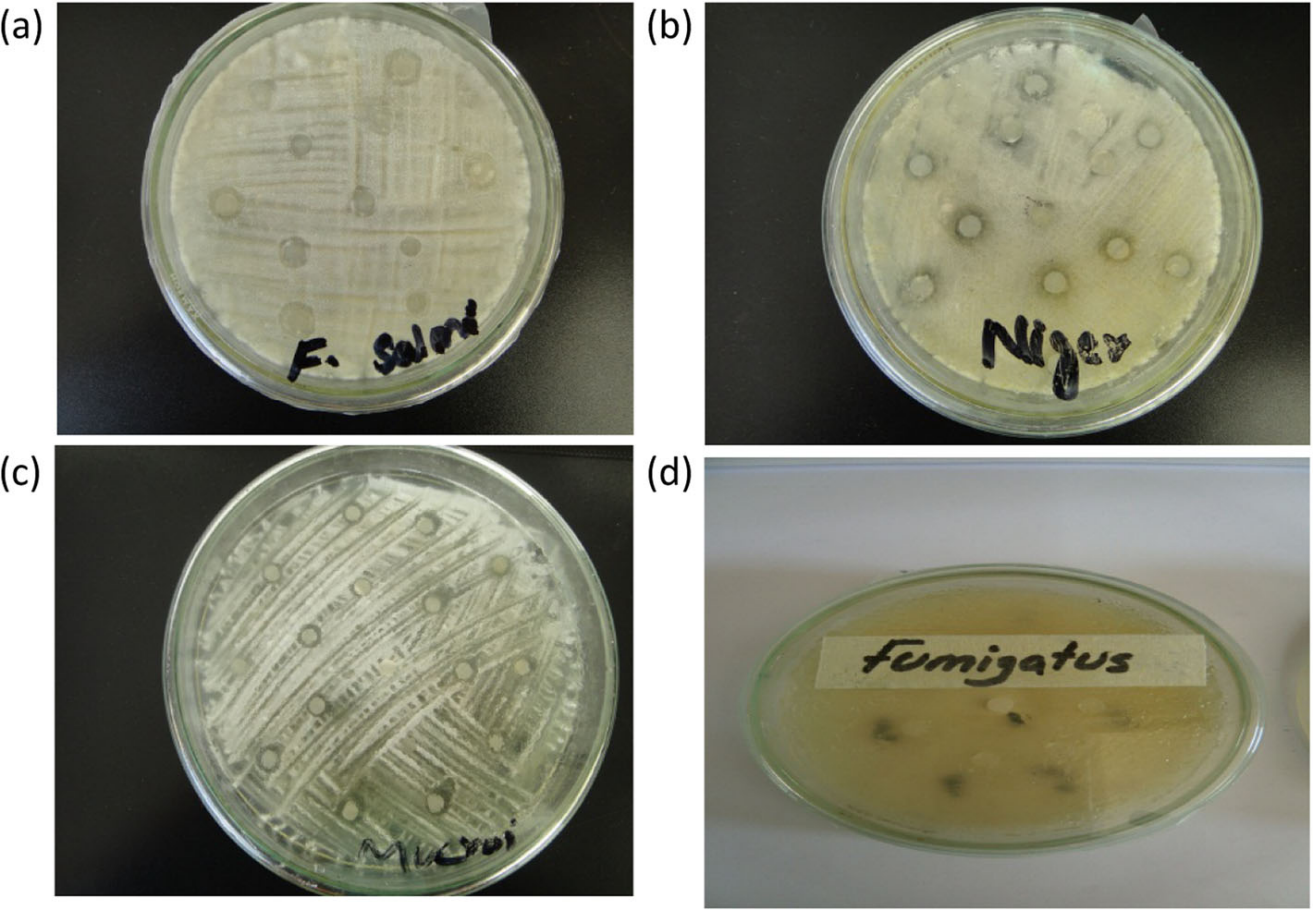
Antifungal activity images of *A. jacquemontii* extracts against **a** *F. soloni* **b** *A. niger* **c** *M. specie* **d** *F. fumagatus*

The sensitivity of each test sample was evaluated against bacterial and fungal strains by disc diffusion method. EtOH and MeOH extracts exhibited highest activity against *M. luteus* with 13.8 ± 1.08 mm zone of inhibition (MIC: 50 μg/ml) and 13 ± 0.15 mm zone (MIC:50 μg/ml) followed by MeOH and Ace+EtOAc with 11.7 ± 1.42 (MIC: 75 μg/ml) and 11 ± 0.51 mm (MIC: 75 μg/ml) zone of growth inhibition against *B. septic,* respectively (Table 2 and Fig. 5). From the susceptibility results against fungal strains, MeOH extract exhibited highest sensitivity against *F. flavus*and *M. specie*with 10.0 ± 0.11 mm and 9.7 ± 0.41 mm growth inhibition zones, respectively. While MeOH+ChCl_3_ exhibited 9.8 ± 0.71 mm growth inhibition zone against *F. solani* followed by MeOH extract with 9.5 ± 0.11 mm inhibition zone (Table 3 and Fig. 6).

**Table 2:** Antibacterial activity of *A. jacquemontii* extracts against Gram-positive and Gram negative bacteria

Microorganism	Diameter of growth inhibition zone (mm)											
	n-Hex	CHCl_3_	Ace	EtOAc	EtOH	MeOH	EtOH+ CHCl_3_	MeOH+ CHCl_3_	Ace + EtOAc	EtOH+ EtOAc	MeOH+ EtOAc	Cef/Rox
*E. aerogens*	--	6.5 ± 0.40^d*^	--	--	--	--	6.5 ± 0.43^d*^	--	--	--	--	24 /22
*S. typhimurium*	7.0 ± 0.92^d*^	--	--	9.0 ± 1.19 ^a*^	9.5 ± 0.99 ^d*^	--	--	8.0 ± 0.71 ^c*^	9.0 ± 1.28 ^c*^	7.0 ± 1.31^d*^	--	18/20
*M. luteus*	--	--	--	^1^13.8 ± 1.08^a*^	--	^2^13 ± 0.15^a*^	--	9.5 ± 1.55 ^c*^	--	--	6.5 ± 0.13^d*^	15/20
*B. bronchiseptica*	9.0 ± 0.67^d*^	8.0 ± 0.51^e*^	^7^10.0 ± 0.46^b*^	9.5 ± 0.38^b*^	^9^10 ± 0.73^b*^	^3^11.7 ± 0.51^a*^	^8^10. ± 0.61 ^c*^	--	^4^11 ± 1.42 ^b*^	^6^10 ± 1.51^c*^	^5^11 ± 0.46 ^b*^	22/20
*S. aureus*	--	8.0 ± 0.57^d*^	--	6.9 ± 1.56^c*^	--	--	7.0 ± 0.58^f*^	--	9.7 ± 0.57 ^b*^	--	7.0 ± 0.59^f*^	18/15

Data Values are presented as mean + SD (*n* = 3)

--: no zone of inhibition, LSD all pair wise comparison Alpha 0.05 (a*, b*, etc.) in which the means are not significantly different from one a another

Positive control = Cef (Cefixime) and Rox (Roxithromycin), Negative control = DMSO (no zone of inhibition)

MIC: ^1^ = 50 μg/ml, ^2^ = 50 μg/ml, ^3^ = 75 μg/ml,^4^ = 75 μg/ml,^5^ = 75 μg/ml,^6^ = 75 μg/ml,^7^ = 75 μg/ml,^8^ = 75 μg/ml,^9^ = 75 μg/ml

**Table 3:** Antifungal activity of *A. jacquemontii* extracts against five fungal strains

Microorganism	Diameter of growth inhibition zone (mm)											
	n-Hex	CHCl_3_	Ace	EtOAc	EtOH	MeOH	EtOH+ CHCl_3_	MeOH + C CHCl_3_	Ace + EtOAc	EtOH+ EtOAc	MeOH+ EtOAc	Terbinafine
*F. fumagatus*	--	--	--	--	--	--	--	--	--	--	--	10 ± 0.71
*M. specie*	--	7.5 ± 0.70 ^b*^	6.25 ± 0.70 ^cd*^	6.2 ± 0.76 ^cd*^	--	9.7 ± 0.41^a*^	5.7 ± 0.75 ^cd*^	--	--	5.5 ± 0.70^e*^	7 ± 0.706^a*^	15 ± 0.77
*A. niger*	--	7.0 ± 0.71^a*^	7.5 ± 1.41^ab*^	--	--	7.7 ± 0.71^a*^	6.5 ± 0.71 ^c*^	06 ± 0.71^c*^	6.7 ± 0.71^bc*^	6.25 ± 0.70^c*^	6.2 ± 0.71^c*^	11 ± 0.56
*F. soloni*	--	--	7.0 ± 0.71^b*^	--	--	--	6.2 ± 0.71^c*^	9.8 ± 0.71^a*^	--	--	--	22 ± 0.81
*F. flavus*	--	--	--	--	--	9.5.0 ± 0.11^a*^	8.7 ± 1.96^b*^	--	--	--	--	12 ± 0.45

Values are presented as mean + SD (*n* = 3)

--: no zone of inhibition, LSD all pair wise comparison Alpha 0.05 a*, b*, etc.in which the means are not significantly different from one another

Positive control = Terbinafine, Negative control = DMSO (no zone of inhibition)

### SRB cytotoxic activity against DU-145, & HL-60

The cytotoxic potential of *A. jaquamontii* tuber extract against the cancer cell lines is presented in Table 4. The heights cytotoxity showed by the n-Hex extract against the DU-145 prostate cancer cell line the ED_50_ as (45.11 μg/mL). EtOAc and MeOH extracts also showed good cytotoxity against the DU-145 with ED_50_ valve 52.2 μg/mL and 66.2 μg/mL, respectively. According to the HL-60 human leukemia cancer cell lines the ED_50_ valve of n-hex extract was 55.2 μg/mL followed by MeOH extract (ED_50_ 66.2 μg/mL). The n-Hex and ChCl_3_ extracts was found to be 34.01 μg/mL and 44.87 μg/mL, respectively in chemo-preventive bioassay by NF-kB and MTP potential.

**Table 4:** Cytotoxic potential of *A. jacquemontii* extracts against DU-145 prostate cancer, HL-60 human leukemia cell lines

Sample code	DU-145		HL-60	
	% survival at 20 μg/mL	ED _50_ μg/mL	% survival at 20 μg/mL	ED_50_ μg/mL
n-Hex	98.2 ± 2.1	45.11	92.6 ± 0.8	55.2
CHCl_3_	68.7 ± 1.5	N/A	52.8 ± 1.7	N/A
Ace	85.3 ± 0.8	N/A	58.1 ± 0.6	N/A
EtOAc	88.2 ± 1.9	52.2	50.6 ± 1.2	N/A
EtOH	45 ± 0.5	N/A	66.2 ± 1.4	N/A
MeOH	89.4 ± 1.5	66.24	86.7 ± 0.8	65.3
EtOH+ CHCl_3_	75.6 ± 1.6	N/A	54.2 ± 0.9	N/A
MeOH+ CHCl_3_	66.3 ± 0.8	N/A	71.4 ± 0.3	N/A
Ace + EtOAc	45.6 ± 1.7	N/A	25.7 ± 1.4	N/A
EtOH+ EtOAc	23.2 ± 0.7	N/A	60.7 ± 0.8	N/A
MeOH+ EtOAc	22.7 ± 1.4	N/A	54.4 ± 0.7	N/A

All data means showed in triplicate. ± means standard error

ED _50_ = concentration at 50% inhibition

Taxol used as a positive control

*N/A* not active

## Discussion

Phytochemicals participate actively in the treatment of various ailments and are considered as significant part of both traditional and modern system of medicines. Polarity dependent extract efficiency variation was observed which signifies the impact of solvent nature on extraction of phytochemicals. The polar solvents tended to extract at higher rate probably due to the presence of polar phytochemicals that are radically available in the water environment (the most powerful polar solvent). It has also been postulated that highly delocalized electrons make most of the plant components highly polarized [19].

Phenols are the compounds that bear a hydroxyl group on its aromatic hydrocarbon ring. The recognition of high concentration of phenolic and flavonoid contents in EtOH and MeOH extracts, respectively of *A. jaquamontii* clues that this plant has significant antioxidative potential. Polar solvents showed better recovery of phenolics and flavonoids due to their ionic nature that support solubility of phenolics and flavonoids. Phenolic compounds are major component of non-enzymatic antioxidant defense system of the cell. This property is due to unique structure of phenols and flavonoids that support them to sequent free radicals [20]. Phenolic compounds also possess multiple biological significance such as antitumor, antimutagenic and antibacterial properties and these activities might be related to their antioxidant activities [21]. The biochemical processes inside the cell produce singlet oxygen and free radical which are lethal to the cell. Such compounds save the cells from these threats [22].

A single assay cannot fully predict the rationality behind antioxidant and radical scavenging activities. Therefore multiple assays are employed that cumulatively describe potential of plant extract as antioxidant. The antioxidant activities of extracts were evaluated using DPPH free radical scavenging assay, total reducing power and total antioxidant capacity assays. The reported DPPH quenching potential of EtOH extract of *A. jaquamontii* was significantly (*P* < 0.05) higher than reported earlier [7, 10]. Other extracts also showed significant DPPH based free radical scavenging activity. However the activity decreased as non-polar solvent was mixed with polar one. A positive correlation was confirmed between TPC, TFC and DPPH scavenging potential of MeOH and EtOH extracts of *A. jaquamontii* which is consistent with previously documented positive connectivity between TPC, TFC and quenching potential. DPPH is nitrogen centered free radical that becomes a stable diamagnetic molecule upon acceptance of an electron or hydrogen radical by donor antioxidant which can be quantitatively measured by changes in absorbance. Reactive oxygen species (ROS) are generated mostly as by-products during mitochondrial electron transport and other metabolic reactions. In addition, ROS are formed as necessary intermediates of metal catalyzed oxidation reactions. These free radicals attack not only nucleic acid bases but also deoxyribosyl backbone of DNA causing genotoxicity and ultimately mutations [9, 23]. Innumerable phytoconstituents (Antioxidants) both non enzymatic and enzymatic perform their part in the blockade by means of scavenging or prevention of generation of ROS. Antioxidant activity determined in DPPH and NBT assays showed 64.16 ± 0.19% and 62.16 ± 0.17%, respectively by *A. jaquamontii* extract.

The phosphomolybdenum based total antioxidant capacity of the extracts involves the reduction of Mo (VI) to Mo (V) and subsequent formation of phosphomolybdate complex. The total antioxidant capacity of the extracts attributes to the total phenolic and flavonoid contents [24, 25]. Phenolic components, such as phenolic acids or phenolic diterpenes, show promising contributors towards antioxidant potential in various medicinal plants. The results strongly correlate the literature where highest antioxidant activities as well as elevated phenolics and flavonoids have been observed in polar extracts including methanol and ethanol. Therefore it can be suggested that active compounds having antioxidant activities are quite polar in nature. Another mode of reduction of free radicals is chelation of metal ions with chelating agents. The transition metal ion Fe^2+^ possesses the ability to reduce the formation of free radicals by gain or loss of electrons. The compounds capable of reducing ferric (Fe^3+^) to ferrous (Fe^2+^) depict their reducing power potential which can be observed by the formation of prussian blue-colored complex when FeCl_3_ is added in reaction mixture [2]. Higher absorbance signifies higher reduction potential [26]. The methanol extract of leaves of *A. jaquamontii* have shown 58% at 100 μg/mL chelating efficiency and polar fractions demonstrated more antioxidant capacity than other fractions [27].

Brine shrimp assay is used to determine cytotoxic activity of sample [28]. This assay has been widely used as prescreening test i.e. antimicrobial, antitumor, antimalarial, insecticidal, etc. Mortality rate of brine shrimps decreased with the concentration of the extract and degree of lethality was perceived to be concentration dependent. The larvicidal property of n-Hex extract of *A. jaquamontii* might be attributed to the presence of some cytotoxic constituents which require bioactivity guided isolation and assessment against further cancer cell lines.

Protein kinase inhibitors depict a distinct class of oncogenic kinase inhibitors. Protein kinases are responsible for the phosphorylation of proteins on serine/threonine and tyrosine residues which are involved in the major regulatory mechanisms in biological processes including apoptosis, cell proliferation, cell differentiation, and metabolism. Occurrence of genetic alterations in early tumor formation can lead to phosphorylation deregulation associated with these pathways and ultimately procreation of cancer. In this regard researchers around the world have shown interest in the identification of kinase inhibitors [29]. Aerial hyphae formation of *Streptomyces* sp. requires protein kinase activity and a variety of kinase inhibitors are able to block this process [30]. The kinase inhibitory values, in this study, are lower than the hyphae formation inhibition values (21 mm clear zone of inhibition at 80 μg/disc) exhibited by critinin isolated from soil filamentous fungus *Penicillium* sp. H9318. It can be inferred that n-Hex extract of *A. jaquamontii* might have potential to bind allosterically with active or inactive site of different kinases and inhibits the progression of cancer. Identification of kinase inhibitors may lead to the development of new anticancer drugs. Deregulation of protein kinases is a key factor that play vital role in the pathogenesis of disease. Protein kinase inhibitors block the aerial hyphae formation of *Streptomyces* sp. [8, 16, 29].

The worldwide dispersion of leishmaniasis and development of resistance against first line therapy demands new therapies for the eradication of this disease [10]. The antileishmanial potential of ChCl_3_ and MeOH extracts (LC_50_ 12.87 and 13.47 μg/ml, respectively) of *A. jaquamontii* was considerably lower LC_50_ than reported earlier [31]. These results are in agreement with previous studies regarding the use of comparatively polar solvents for better extraction of antimicrobial compounds.

The extracts when analyzed against different strains of bacteria, Gram positive strain appeared more susceptible to the inhibitory effect than Gram negative strains. The presence of an extra covering outside the Gram negative bacteria may resist the drug/extract from being entered into the cell. The results are in good accordance with another study in which MeOH extract of *Arisaema utile* showed strong antimicrobial effects against the all tested microorganisms [32]. Distribution of activity in less polar fractions indicates that active compounds of the plant are non polar to slightly polar in nature. The antimicrobial activity might be strictly attributed to the synergistic/antagonistic effect of multiple phytochemicals in the composition of a sample. The current results confirm the presence of highly polar to medium polar compounds in *A. jacquemontii*, which can be a remarkable source of antibacterial agents. This might be due to distribution of active component in specific extract depending upon its solubility nature. *A. jaquamontii* antibacterial activity revealed that n-Hex and EtOAc soluble fractions significantly inhibited the bacterial growth while other showed moderate activity (6,33) The natural bactericidal agents are considered safer in their usage as compared to synthetic and semi synthetic antimicrobial agents. Similarly results also reinforce the substantiation that MeOH might be an effective solvent for enhanced and efficient extraction of antimicrobials [33, 34]. Such studies inspired the scientist to identify other bioactive compound through isolation.

According to SRB cytotoxic effects, *A. jquamontii* is highly active against DU-145, and HL-60 cencer cell lines. n-Hex extract showed higher cytotoxity against both cancer cell lines while other extracts did not show remarkable activity against these cancer cell lines. According to literature lectin, a phytocomponent of *A. jacquemontii* inhibit in vitro cytotoxity against PC-3 as (30%). While anther species of *A. helleborrifolium* showed an inhibitory effect of MTP and the cytotoxicity against HOP-62, as (95%) and HCT-15 as (92%) [32]. *A. jaquamontii* extracts have been proven for immune modulating potential by T-cell and B-cell proliferation that is due to decrease in acid phosphatase and alkaline phosphatase activities [27, 35]. The NF-κB signaling has a potential application for the prevention or treatment of cancer. Mammalian cells express NF-kB that is a key nuclear transcription factor.

## Conclusion

The phytochemical and biological potential of *A. jacquemontii* tuber extracts prepared in different solvent system indicate that selection of solvent affect on phytochemical and biological activities. Polar solvent extracts might be the potential source of phytochemicals provoking highly significant antioxidant, cytotoxic, anticancer and protein kinases inhibitor. The study also indicates that methanol and ethanol extracts of *A. jaquemontii* tuber have high antioxidant potential and radical scavenging activity. Remarkable cytotoxic and antimicrobial activities were best observed in the n-hexane and methanol extracts, respectively. Notable activity by n-hexane extract against DU-145 prostate cancer cell lines and HL-60 human leukemia cancer cell lines highlight that this plant specie has anticancer agents. In conclusion the species of Ariseama possess height cytotoxity against the different cancer cell lines and can be used for chemo preventive agent. Tuber of *A. jaquamontii* may be considered as a rich source of potential secondary metabolites with significant biological activities and act as lead source of potent pharmaceuticals.

## Data Availability

The datasets supporting the conclusions of this article are included within the article.

## Abbreviations

DMSO: Dimethyl sulfoxide
DPPH: 2, 2-diphenyl-1-picrylhydrazyl
DU-145: Prostate cancer cell lines
FRSA: Free radical scavenging activity
HL-60: Human promyelocytic leukemia cell line
IC_50_: 50% inhibitory concentration
LC_50_: Lethal concentration causing 50% mortality
MIC: Minimum inhibitory concentration
MTP: *Mitochondrial transmembrane potential*
NF-kB: *Nuclear factor kappa-B*
SRB: *Sulphorhodamine B*
TAC: Total antioxidant capacity
TFC: Total flavonoid contents
TPC: Total phenolic contents
TRP: Total reducing power
ZOI: Zone of inhibition
